# Low Vitamin D Levels Are Associated with Higher Opioid Dose in Palliative Cancer Patients – Results from an Observational Study in Sweden

**DOI:** 10.1371/journal.pone.0128223

**Published:** 2015-05-27

**Authors:** Peter Bergman, Susanne Sperneder, Jonas Höijer, Jenny Bergqvist, Linda Björkhem-Bergman

**Affiliations:** 1 Department of Laboratory Medicine, Division of Clinical Microbiology, Karolinska Institutet and Karolinska University Hospital, Huddinge, Stockholm, Sweden; 2 ASIH Stockholm Södra, Långbro Park, Palliative Home Care and Hospice Ward, Bergtallsvägen, Älvsjö, Sweden; 3 Institute of Environmental Medicine, Unit of Biostatistics, Karolinska Institutet, Stockholm, Sweden; 4 Department of Oncology/Pathology, Karolinska Institutet and Stockholms Sjukhem, Unit for Palliative Care, Stockholm, Sweden; Tokyo Metropolitan Institute of Medical Science, JAPAN

## Abstract

**Background:**

Vitamin D deficiency is common among palliative cancer patients and has been connected to an increased risk for pain, depressions and infections. Therefore we wanted to test the hypothesis that low 25-hydroxyvitamin D (25OHD) levels are associated with higher opioid dose, higher infectious burden and impaired quality of life in palliative cancer patients. The secondary aim was to investigate the association between 25OHD-levels and survival time.

**Method:**

In this prospective, observational study in palliative cancer-patients (n = 100) we performed univariate and multiple linear regression analysis to assess the association of 25OHD levels with opioid dose, infectious burden (antibiotic consumption), quality of life (Edmonton Symptom Assessment Scale, ESAS) and survival time, controlling for potential confounding factors.

**Results:**

The median 25OHD level was 40 nmol/L (range 8-154 nmol/L). There was a significant association between 25OHD levels and opioid dose, beta coefficient -0.67; p=0.02; i.e. a low 25OHD level was associated with a higher opioid dose. This association remained significant after adjustment for stage of the cancer disease in a multivariate analysis, beta coefficient -0.66; p = 0.04. There was no association between 25OHD levels and antibiotic use or quality of life. Univariate cox regression analysis showed a weak correlation between survival time and 25OHD levels (p<0.05). However, decreased albumin levels and increased CRP levels were superior markers to predict survival time; p<0.001 for both analyses.

**Conclusion:**

Low 25OHD-levels are associated with increased opioid consumption in palliative cancer patients. Future interventional studies are needed to investigate if pain can be reduced by vitamin D supplementation in these patients. In addition, this study confirms previous findings that low albumin and increased CRP levels are useful markers for survival time in palliative cancer patients.

## Introduction

Palliative cancer patients often suffer from pain and depression. Bacterial infections are also a common problem that impair the quality of life and shorten lifespan in these patients. Several observational studies show that cancer patients generally have lower vitamin D levels than healthy controls [1–4]. Since cancer is often connected with both depression and pain it is of notable interest that Vitamin D supplementation may be beneficial in depressive disorders [4,5] and that low vitamin D levels are associated with increased risk of pain [6–9]. In addition, vitamin D affects the human immune system in several ways, e.g. by inducing the synthesis of antimicrobial peptides at mucosal surfaces and in immune-cells [10]. Several randomized controlled trials (RCTs) show beneficial effects of vitamin supplementation against infections [11–13]. Moreover, observational studies have shown that low vitamin D-levels are associated with increased all-cause mortality [14,15]. In a meta-analysis of 159 RCTs it was shown that treatment with vitamin D_3_ was associated with decreased mortality, and especially mortality caused by cancer [16].

Vitamin D is synthesized in the skin under influence of UVB-light and further hydroxylated in two steps. The first hydroxylation occurs in the liver to the proform 25-hydroxyvitamin D (25OHD), whereas the second step generates active vitamin D (1,25 (OH)_2_ vitamin D) and occurs via 1-alpha hydroxylase (CYP27B1), which is expressed in kidney cells, but also in many other cells in the body [17]. Active vitamin D binds to the vitamin D receptor (VDR), which regulates a large number of genes [18]. The half-life of 25OHD is about 3 weeks, whereas 1,25-dihydroxyvitamin D only has a half-life of about 4 hours [19]. Generally, systemic levels of the more stable 25OHD are considered to reflect vitamin D-status in the individual patient [17]. According to recommendations from the Institute of Medicine in the U.S., serum levels of 25OHD below 50 nmol/L are considered to be insufficient [20].

Given that palliative cancer patients often suffer from pain, infections and depression there is a solid rationale to study the relationship between 25OHD levels and these outcomes. Therefore we designed a prospective and observational study at a palliative ward in Stockholm, Sweden. The primary aim of this study was to test the hypothesis that low 25OHD levels are associated with higher opioid dose, higher infectious burden and impaired quality of life in palliative cancer patients. As a secondary aim, we also wanted to investigate if there was an association between 25OHD levels and survival time and compare with the well-established markers albumin and CRP, often used in clinical practice to estimate survival time in cancer patients [21]. This pilot-study was performed to obtain relevant baseline information for a future interventional trial testing the hypothesis that vitamin D can reduce pain, infections and depression among palliative cancer patients.

## Materials and Methods

### Study population

This was a prospective, observational study to investigate the association between 25OHD levels and opioid dose, infections and quality of life. Data was collected between April 2014—January 2015. One hundred palliative cancer patients were recruited from ASIH Stockholm Södra, Långbro Park Palliative Home Care Team and Hospice Ward. Written informed consent was obtained from all participants prior to inclusion. After study inclusion a serum sample was drawn and 25OHD, albumin and CRP was measured and clinical data was extracted from the medical records. Levels of 25OHD in serum were analyzed by chemiluminescence immunoassay (CLIA) on a LIAISON-instrument (DiaSorin Inc, Stillwater, MN, USA,) detectable range 7.5–175 nmol/L, CV 2–5% at the Department of Clinical Chemistry, Karolinska University Hospital. Infectious burden was calculated as antibiotic consumption 3 months before study inclusion. A ratio of ‘days with antibiotics’ compared to ‘days without antibiotics’ was calculated for each patient. Opioid dose at the day of inclusion was recorded and translated to corresponding fentanyl dose per hour. The self-assessed quality of life was recorded with the Edmonton Symptom Assessment Scale (ESAS) [22] that is routinely monitored every second week in all patients at ASIH Långbro Park. In this scale 10 different parameters are assessed, where ‘quality of life’ is one such parameter. The ESAS-scale ranges from 0–10, where the number 10 is the ‘worst possible quality of life’ and 0 represents a ‘good quality of life’ [22]. In addition, the survival time was registered for all patients in the study.

The raw data is available from the corresponding author upon request.

### Ethic Statement

The study was approved by the local Ethical Committee at Karolinska Institutet, Stockholm, Sweden (Dnr: 2014/455-31/4) and was performed in accordance with the declaration of Helsinki.

### Statistical analysis

Statistical tests were performed using Stata v. 13 and GraphPad Prism v. 6.00. Values of p < 0.05 were considered statistically significant. When analyzing the outcomes of opioid doses, infectious burden and quality of life (ESAS), linear regression analysis was used, while the survival analysis was conducted using Cox proportional hazard regression. Since quality of life, measured by ESAS, is not a continuous variable, we assessed the validity of the linear regression using an ordinal logistic regression, after trichotomizing the original variable into the categories 1–4, 5–7 and 8–10. Since there were some missing values in the variables “ESAS”, “CRP” and “albumin”, a sensitivity analysis was conducted whenever these variables were included, using multiple imputation.

The parameters “25OHD-values”, “age” and “survival time” were not normally distributed as determined by the Komolgorov-Smirnov test. Thus, we presented median and range values in the result-section. Also, due to the skewed distribution, the non-parametric Mann-Whitney U-test was applied when comparing 25OHD-levels between patients who survived or died during the study-period as well as between patients with 25 OHD levels above or below 50 nmol/L.

Since this was a pilot-study with the aim to obtain data for a future interventional study, no power calculation was carried out.

## Results

### Baseline demography

One hundred palliative cancer patients were included in the study, 57 women and 43 men. The median age was 71 years (range 17–93). Seventy-one patients died during the study and 29 were still alive at the end of the study. The median survival time among patients who died was 23 days. All patients hade incurable cancer and the diagnoses were: gastrointestinal cancers (n = 20), lung cancer (n = 19), gynecological cancer (n = 13), pancreatic cancer (n = 13), breast cancer (n = 10), prostate cancer (n = 7), hematologic malignancy (n = 5), head-neck cancer (n = 3), malignant melanoma (n = 3), cholangiocarcinoma (n = 2), brain tumor (n = 2) and other cancers (n = 3).

The median 25OHD level in the cohort was 40 nmol/L (range <8–154 nmol/L). Patients who survived at the end of the study had a significantly higher 25OHD level (median 50 nmol/L) than those who died during the study period (median 36 nmol/L; p = 0.013, Mann-Whitney U-test).

Patients with 25 OHD levels < 50 nmol/L (n = 65) had a significantly higher opioid dose than those with 25 OHD levels above 50 nmol/L (n = 35); mean 74 μg fentanyl/h compared to 43 μg fentanyl/h (Mann Whitney U-test p = 0.04). There was no difference in ESAS score or antibiotic consumption among patients below or above 50 nmol/L.

### Primary aim:25OHD-levels in relation to opioid dose, antibiotic use and quality of life

**Fig 1:**
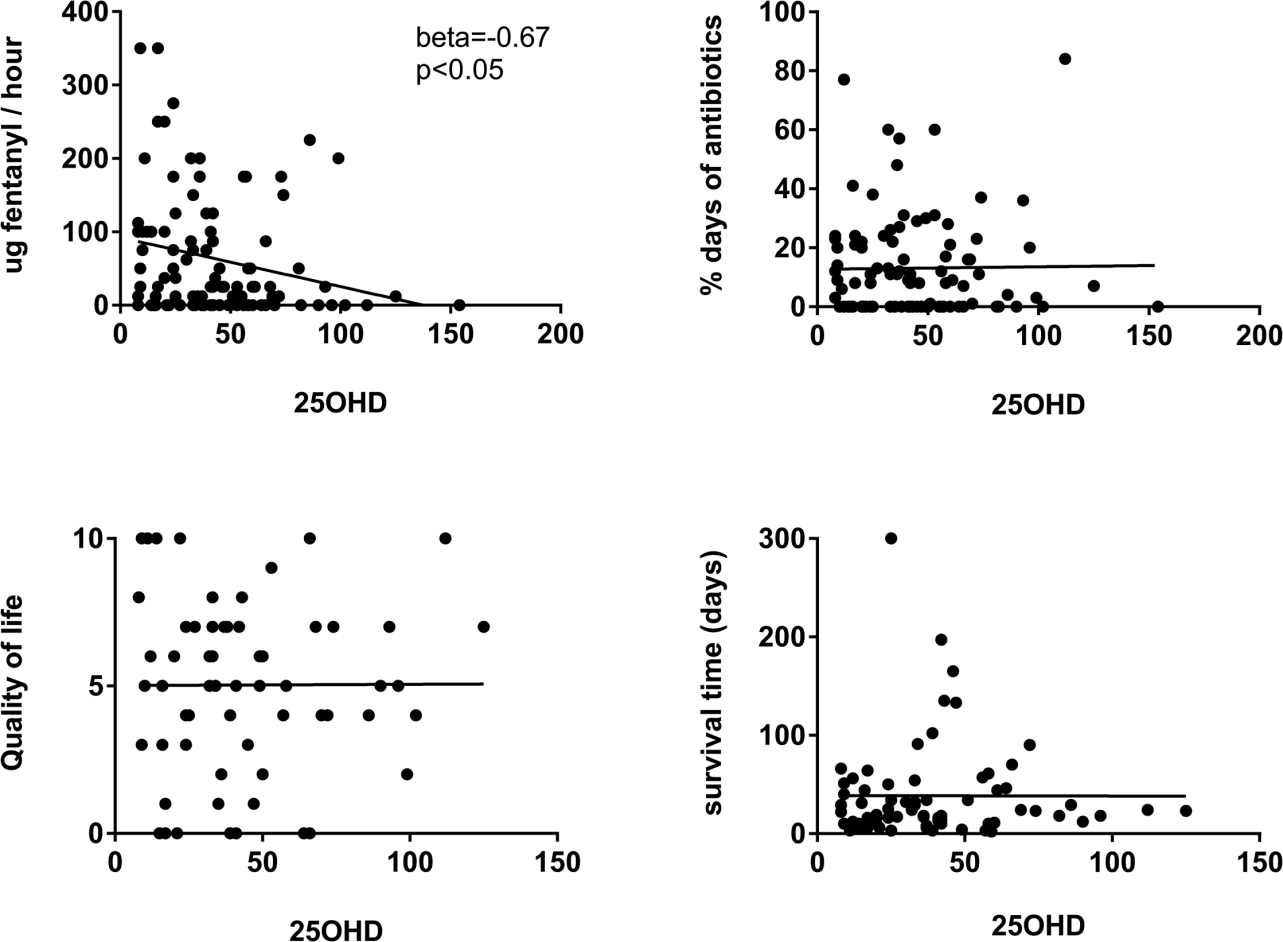
Linear regression analysis of 25-hydroxyvitamin D levels (25OHD) and different parameters. The 25OHD levels in 100 palliative cancer patients showed a significant correlation with opioid dose, expressed as fentanyl μg/hour, but not with infectious burden expressed, as percentage antibiotic consumption per day for 3 months, or self-assessed quality of life expressed as ESAS (numeric scale 1–10 were 10 is worst possible quality of life) or survival time (n = 71).

To investigate a possible association between 25OHD levels and the different parameters studied, univariate linear regression was performed (Table 1, Fig 1). There was a significant association between 25OHD level and opioid dose; beta coefficient = -0.67 (p = 0.02); i.e. a low 25OHD level was associated with a higher opioid dose. The beta coefficient indicates that for every nmol increase in 25OHD level the opioid dose decreased with 0.67 μg/fentanyl/hour. There was no association between 25OHD levels and antibiotic use or quality of life, measured by ESAS. A sensitivity analysis with multiple imputation, gave substantially the same results, as did the analysis using ordinal logistic regression (data not shown).

**Table 1:** Univariate analysis with linear regression in 100 palliative cancer patients.

Dependent variable	beta coefficient	p-value	95% CI
**Opioid (μg fentanyl / hour)**	**-0.669**	**0.022**	**-1.240; -0.098**
**ESAS (quality of life)**	**0.00044**	**0.973**	**-0.026; 0.027**
**Infections (% days w antibiot.)**	**0.000075**	**0.904**	**-0.001; 0.001**
**Survival time (days)**	**-0.005**	**0.983**	**-0.470; 0.460**

The 25-hydroxyvitamin D (25OHD levels) is the independent variable and the dependent variable is opioid dose (μg fentanyl / hour), ESAS as a marker for quality of life on a numeric scale 1–10 where 10 is worst, infectious burden (% of days with antibiotics) and survival time (n = 71).

Next, we tested whether the association between 25OHD levels and opioid-use remained after adjustment for stage of the disease by applying multiple linear regression analysis (Table 2). In an attempt to determine “stage in the disease” we used the parameters CRP, albumin and time to death. The association between 25OHD levels and opioid use remained significant also after adjusting for these factors, beta coefficient = -0.66 (p = 0.04). However, when age was included in the multivariate model, the relation between 25OHD levels and opioid use was no longer significant. This suggests that age has a major impact on the relation between vitamin D levels and opioid use (Table 2). Notably, in this study cohort, higher age was associated with increased 25OHD levels (beta coefficient = 0.05; p = 0.02, linear regression) in contrast to population based studies on healthy individuals [23]. Higher age was also associated with a lower opioid dose (beta coefficient = -1.4; p<0.01, linear regression).

**Table 2:** Multiple linear regression model to investigate the association between opioid dose (μg fentanyl / hour) and 25-hydroxvitamin D levels with adjustment for different confounding factors.

Adjustments	coefficient	p-value	95% CI
**Sex, albumin, CRP, time to death / survival time**	**-0.659**	**0.041**	**-1.292; 0.027**
**Age, sex, albumin, CRP, time to death / survival time**	**-0.459**	**0.166**	**-1.112; 0.195**

Opioid dose is the dependent variable.

### Secondary aim: Survival Analysis

**Fig 2:**
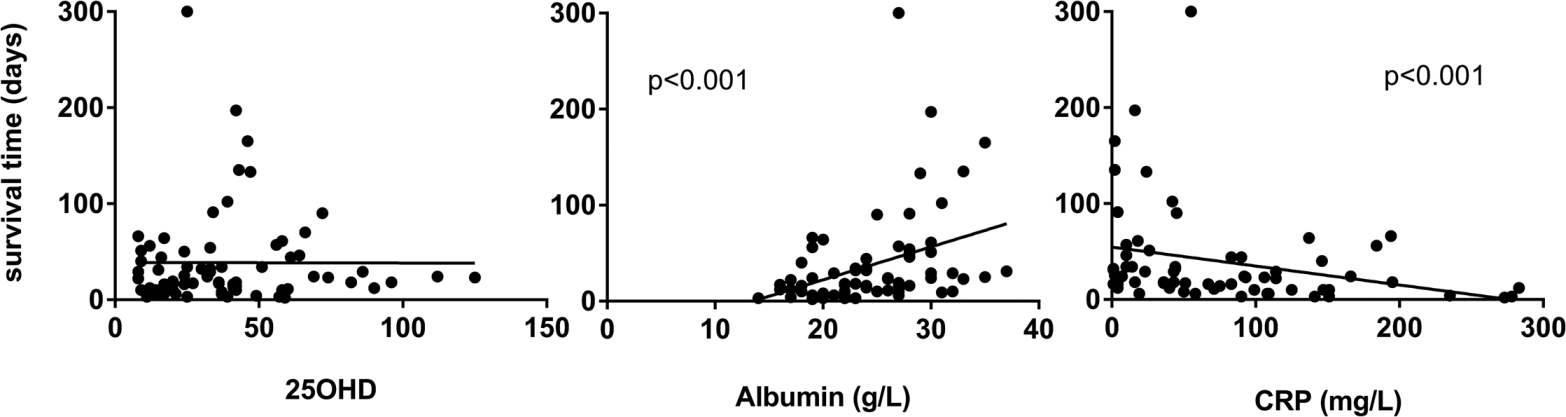
Cox regression analysis of survival time and different biochemical parameters. Survival times in 71 palliative cancer patients were significantly associated with albumin and CRP levels but not with 25-hydroxyvitamin D levels (25OHD). Cox regression showed hazard ratio (HR) 0.87 (95% CI and 0.84–0.92; p<0.001) for albumin, HR 1.005 (95%CI 1.002–1.007; p<0.001) for CRP and HR 0.99 (95%CI 0.989–0.999; p = 0.026) for 25OHD-levels.

To investigate a possible association between 25OHD levels and survival time, univariate Cox regression was performed (Fig 2). An association was noted between 25OHD levels and survival time; HR 0.99 (95% CI 0.989–0.999; p = 0.026), suggesting that an increase of vitamin D with 1 unit (1 nmol/L) would lower the risk of death (hazard) with 1%. To put this finding in the context of known and well described biomarkers for survival time, Cox regression analysis was also performed for albumin and CRP in relation to survival. As expected, albumin was strongly associated with survival time (hazard ratio (HR) 0.87; 95% CI and 0.84–0.92; p<0.001), whereas CRP levels exhibited a weaker, but statistically significant, association with survival time (HR 1.005; 95% CI 1.002–1.007; p<0.001). Again, a sensitivity analysis with multiple imputations, did not alter the results in any substantial way.

Next, we performed a multivariate analysis in which we wanted to study if the association between 25 OHD and survival time was still present after controlling for albumin and CRP. In this model the association between 25OHD and survival time was no longer significant, (HR 0.998; 95% CI 0.989–1.008; p = 0.75).

Taken together, these data suggest that 25OHD-levels are associated with survival, but albumin has a significantly better precision as a marker in clinical practice.

## Discussion

In this study we show that there was a significant correlation between 25OHD levels and opioid dose, where a lower 25OHD level was associated with a higher dose of opioids. Patients with 25 OHD levels < 50 nmol/L, defined as vitamin D deficiency [20], had significantly higher opioid dose than those with 25 OHD levels above 50 nmol/L. The association between 25 OHD and opioid dose remained significant after adjustment for sex and stage of the disease (surrogate markers used: CRP, albumin and survival time / time to death). However, when age was added to the multivariate regression model, the association between 25OHD levels and opioid use disappeared. Consistent with this result, our data show that vitamin D-levels increased with age and also that older patients used less opioids than younger patients. It could thus be argued that the age-factor fully could explain the observed association between vitamin D levels and opioid use. However, a closer look at the data revealed that surviving patients had significantly higher 25OHD levels than those who died during the study. Thus, it is possible that older patients survived longer with their cancer due to their higher vitamin D levels. This interpretation could explain why we observed that older patients had higher vitamin D levels and also consumed less opioids. However, the observational design of this study precludes any definitive conclusions on causality.

The 25OHD levels were generally low among all patients in this study, median 40 nmol/L. This is in accordance with several observational studies that have shown that cancer patients often have lower 25OHD levels than healthy controls [1–4]. If the low 25OHD levels are indirectly caused by cancer, for example by fewer out-door activates and less sun-light exposure during the disease time, or if subjects with low 25OHD levels are more susceptible to cancer disease is not known.

The finding that older patients have higher vitamin D levels is at odds with previous data from large population based studies on healthy individuals, where older patients generally have lower vitamin D levels than younger individuals [23]. The general notion is that elderly people spend less time in the sun, have reduced levels of 7-dehydrocholesterol in the skin and eat less vitamin D containing food and thus end up with lower levels of 25OHD in the circulation [23]. Interestingly, in cancer patients the situation appears to be the opposite, i.e. older patients have higher levels of 25OHD [24,25]. One reason for this paradox could be that patients with a sufficient vitamin D status live longer with their cancers. In fact, this is supported by several prospective studies where a low vitamin D level has been shown to predict a worse outcome and also a shorter life span in patients with several different cancer forms, including colorectal and renal cancer [26–29]. Moreover, several large meta-analyses have found that low vitamin D levels are associated with an increased overall mortality and also with death caused by cancer [14,15,30]. Thus, it is possible that vitamin D has a true effect on pain mechanisms in cancer patients and that the confounding effect of age, can be explained by the fact that patients with a high vitamin D level survive longer with their cancers.

There are several pieces of evidence for a role of vitamin D in pain, both in observational [6–9] and in interventional studies [6,8,31]. From a mechanistic point of view, there are limited data available, but a recent study showed that patients with insufficient 25OHD levels (<50 nmol/L) had more pain and exhibited pathological nerve conduction [32]. Interestingly, vitamin D insufficiency is also associated with peripheral neuropathy and autonomic nerve dysfunction in patients with type 2 diabetes [33]. Finally, vitamin D was recently found to contribute to re-myelination in a rat model of nerve damage [34]. Taken together, there is ample evidence from observational, interventional and experimental studies that vitamin D could have a beneficial role against pain in various settings.

Nevertheless, it can also be argued that vitamin D does not have any direct or causal role in pain. In fact, a recent article suggested that UVB-light has strong analgesic effects by induction of endogenous opioid-like substances (endorphins) in the skin. In this model, vitamin D levels would only serve as a maker for UVB-exposition. Given these novel and exciting data, it is possible that our finding of a clear association between vitamin D levels and opioid use can be fully or partly explained by exposure to the sun and endogenous synthesis of endorphins. In support of this notion, it has been suggested that elderly men have more time to spend outdoor and therefore have higher vitamin D levels [35]. Unfortunately, we have no information on time spent outdoors or on levels of endogenous endorphins in plasma for patients in this study.

There was no correlation between 25OHD levels and quality of life (QoL). Previous reports have shown that vitamin D supplementation may be beneficial in the treatment of depression but the effects seems to be moderate [36] and the results are still inconclusive [5]. In a randomized, placebo-controlled trial in elderly patients (n = 120), a single megadose of vitamin D (300.000 IU as a bolus dose) increased quality of life and reduced pain [37]. However, according to a recent systematic review, the evidence for a link between vitamin D levels and quality of life is weak [36], although these studies did not comprise palliative patients. We have recently shown in a post-hoc analysis from a randomized, placebo-controlled vitamin D study in patients with immunodeficiency that high 25OHD levels >100 nmol/L were correlated to an overall higher assessment of well-being [38]. However, well-being in cancer-patients is highly dependent on burden of symptoms, stage of the disease, prognosis as well as social and psychological factors [39]. If there is an association between 25OHD levels and quality of life the association is probably rather weak and a much larger cohort would be needed to establish such an association. In addition, any future study testing the hypothesis that vitamin D could be beneficial on QoL in palliative cancer patients, should consider to use a validated assessment form, such as EORTC QLQ-C30 [39] to obtain more valid data.

There was a significant association between survival time and decrease in albumin level or increase in CRP levels. Previous studies have also shown an association with elevated CRP levels and decreased albumin levels and a poor prognosis [21]. An association was noted between 25OHD levels and survival time. However, this result should be interpreted with caution since it was an unadjusted comparison in a small study sample. Importantly, the predictive capacity of 25OHD levels on survival time was inferior to albumin in particular, but also to CRP, which both exhibited a significantly higher precision in this respect.

There are several strengths of this study. First, the study cohort is well-defined, thoroughly followed and spans over all ages (17–93 years old) and all cancer-forms. However, this heterogeneity might also be a limitation since possible effects of vitamin D may apply for certain cancers only. Another strength is that the 25OHD levels span over a large interval in the cohort (<8–154 nmol/L). In addition, since most patients were in a late stage of disease and had on average high opioid doses in combination with the fact that a large proportion of the cohort had deficient 25 OHD levels (<50 nmol/L) might also have contributed to the finding of an association.

The major weakness of the study is the observational design, precluding the possibility to draw conclusion on causality. Another weakness is how the infectious burden was related to 25OHD levels in this study. Cancer patients are a very heterogeneous group; e.g. due to their stage of disease and degree of immunosuppression, which affects their susceptibility for infections. Thus, the optimal way to study whether vitamin D could prevent infections in this group would be a longitudinal and interventional study where patients serve as their own controls, before and after initiation of vitamin D supplementation. Further, the use of ESAS-assessment as a marker for quality of life could also be questioned and a more detailed self-assessment of quality of life using a validated questionnaire for palliative cancer patients such as EORTC QLQ-C30 [39] would have been preferable. Another limitation is the relatively small sample size (n = 100) in the study. Finally, it is important to state that this study has a pilot-format and thus lack a *bona fide* sample size estimation. In fact, the study was performed to obtain relevant baseline information for a future interventional trial testing the hypothesis that vitamin D can reduce pain, infections and depression among palliative cancer patients. Therefore, a lack of association in this study could simply be a result of an underpowered sample-size. Nevertheless, not many studies are carried out in terminal or pre-terminal cancer patients and it is often difficult due to ethical and practical reasons to recruit patients to participate in clinical studies if they have a very short time left in life. Therefore, despite the small sample size and lack of power calculation, the study adds relevant information to the field and further supports additional research on the role of vitamin D in palliative cancer patients.

### Conclusions

In conclusion, low 25OHD-levels are associated with increased opioid consumption in palliative cancer patients. Future interventional studies are needed to investigate if pain can be reduced by vitamin D supplementation in this vulnerable group of patients. In addition, this study confirms previous findings that low albumin and increased CRP levels are useful markers for survival time in palliative cancer patients.
